# Minimal domain of bacterial phytochrome required for chromophore binding and fluorescence

**DOI:** 10.1038/srep18348

**Published:** 2015-12-18

**Authors:** Konstantin A. Rumyantsev, Daria M. Shcherbakova, Natalia I. Zakharova, Alexander V. Emelyanov, Konstantin K. Turoverov, Vladislav V. Verkhusha

**Affiliations:** 1 Department of Anatomy and Structural Biology, Albert Einstein College of Medicine, Bronx, NY 10461, USA; 2 Laboratory of Structural Dynamics, Stability and Folding of Proteins, Institute of Cytology, Russian Academy of Sciences, St. Petersburg 194064, Russia; 3 Department of Cell Biology, Albert Einstein College of Medicine, Bronx, NY 10461, USA; 4 Department of Biophysics, Peter the Great St. Petersburg Polytechnic University, St. Petersburg 195251, Russia; 5 Department of Biochemistry and Developmental Biology, Faculty of Medicine, University of Helsinki, Helsinki 00290, Finland

## Abstract

Fluorescent proteins (FP) are used to study various biological processes. Recently, a series of near-infrared (NIR) FPs based on bacterial phytochromes was developed. Finding ways to improve NIR FPs is becoming progressively important. By applying rational design and molecular evolution we have engineered *R. palustris* bacterial phytochrome into a single-domain NIR FP of 19.6 kDa, termed GAF-FP, which is 2-fold and 1.4-fold smaller than bacterial phytochrome-based NIR FPs and GFP-like proteins, respectively. Engineering of GAF-FP involved a substitution of 15% of its amino acids and a deletion of the knot structure. GAF-FP covalently binds two tetrapyrrole chromophores, biliverdin (BV) and phycocyanobilin (PCB). With the BV chromophore GAF-FP absorbs at 635 nm and fluoresces at 670 nm. With the PCB chromophore GAF-FP becomes blue-shifted and absorbs at 625 nm and fluoresces at 657 nm. The GAF-FP structure has a high tolerance to small peptide insertions. The small size of GAF-FP and its additional absorbance band in the violet range has allowed for designing a chimeric protein with *Renilla* luciferase. The chimera exhibits efficient non-radiative energy transfer from luciferase to GAF-FP, resulting in NIR bioluminescence. This study opens the way for engineering of small NIR FPs and NIR luciferases from bacterial phytochromes.

There is a growing demand for the development of near-infrared (NIR) fluorescent proteins (FPs) as genetically encoded NIR probes used in studing metabolic processes noninvasively and deep tissue^1^. NIR light has advantages in penetrating mammalian tissues much deeper than visible light and resulting in less light scattering. Bacterial phytochrome photoreceptors (BphPs) are the most suitable templates for engineering NIR FPs because of their natural NIR-shifted absorbance spectra and the abundance of biliverdin IXα (BV) chromophore in mammalian cells^2^. BV is a tetrapyrrole compound enzymatically produced from heme. BphPs serve as templates for engineering constitutively fluorescent NIR FPs^3^,^4^,^5^,^6^,^7^,^8^, photoactivatable NIR FPs^9^ and NIR reporters of protein-protein interactions^10^,^11^.

Despite having advanced features as deep-tissue fluorescent probes, the best available BphP-derived NIR FPs are ~40% larger than common GFP-like FPs and typically form dimers. These drawbacks originate from the features of natural BphPs such as multidomain organization, the figure-eight knot structure and the dimerizing interface between two BphP monomers^12^,^13^. In BphPs, both PAS (Per-ARNT-Sim) and GAF (cGMP phosphodiesterase/adenylate cyclase/FhlA transcriptional activator) domains are required for BV chromophore binding: the PAS domain contains a Cys residue at the N-terminal extension that covalently binds to BV located in the pocket of the GAF domain^14^. The domain-domain interaction is tightened by the knot structure in which the N-terminus of the PAS domain passes through the loop of the GAF domain^15^. These structural features do not allow for decreasing the size of BphP-based NIR FPs, which limits their range of potential applications^16^.

Notably, cyanobacteriochromes, a subclass of photoreceptors related to phytochromes, consist of only GAF domains, which are able to autocatalytically bind their tetrapyrrole chromophore, called phycocyanobilin (PCB)^17^,^18^. It was shown that GAF domains of several cyanobacteriochromes fluoresce with PCB^19^,^20^,^21^ and that one of them, AM1_1557g2, is able to bind BV^21^.

We hypothesized that by moving the Cys residue from the N-terminal extension of the PAS domain of BphP to its GAF domain^22^ we could combine within a single domain both the covalent binding of BV chromophore and its positioning in a specific pocket. Here, we report a small monomeric NIR FP developed from the GAF domain of the *Rp*BphP1 protein of a gram-negative purple non-sulfur bacterium *Rhodopseudomonas palustris*^23^.

## Results and Discussion

### Engineering of a single-domain near-infrared fluorescent protein

**Figure 1:**
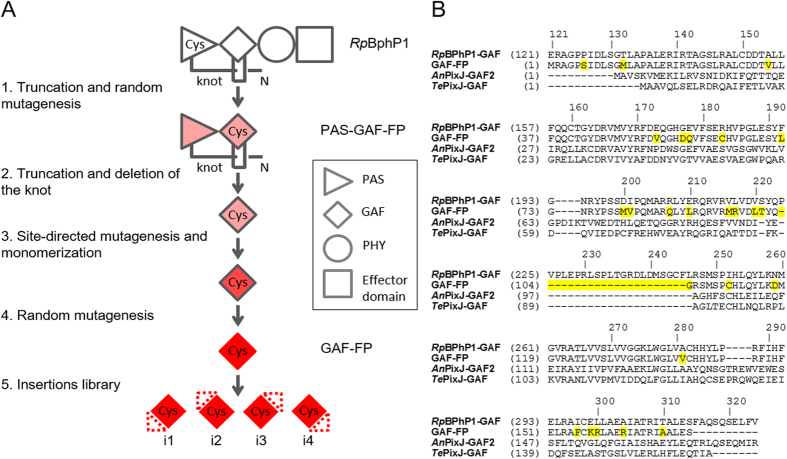
Rational design and molecular evolution of GAF-FP. (**A**) Schematic representation of the directed molecular evolution of natural *Rp*BphP1 resulted in GAF-FP and its small peptide insertion variants. The red color intensity depicts the fluorescence levels of the respective proteins. (**B**) Alignment of the amino acid sequence of GAF-FP with GAF domains of parental *Rp*BphP1 and *An*PixJ and *Te*PixJ cyanobacteriochromes. The GAF-FP residues that differ from those of *Rp*BphP1 are highlighted in yellow. Amino acid numbering follows that of *Rp*BphP1.

We have applied a directed molecular evolution to wild-type *Rp*BphP1 (Fig. 1A). First, we truncated the *Rp*BphP1 template to GAF and PAS domains. We next targeted Asp200 and Ile201 positions (Fig. 1B; amino acid numbering follows that of *Rp*BphP1) in a conservative -197PXSDIP202- amino acid motif using saturated mutagenesis to prevent BV photoisomerization and subsequent non-radiative energy dissipation, thus rendering the protein fluorescent^5^,^8^,^24^. Then we performed random mutagenesis. During screening the bacterial libraries of mutants we found an FP variant, called PAS-GAF-FP, capable of weak fluorescence and at the same time comprising two amino acid substitutions, Cys19Ser and Ile252Cys, of *Rp*BphP1. Interestingly, the absorbance and fluorescence emission maxima of PAS-GAF-FP variant were similar to those found in blue-shifted two-domain (PAS-GAF) iRFPs^8^. The Cys19Ser substitution suggested that this FP variant was no longer able to covalently bind BV in the PAS domain, and possibly a new Cys252 residue became responsible for this function. Our hypothesis was based on the similarity between the position of Cys252 and chromphore-binding Cys residues in the GAF domains of plant and cyanobacterial phytochromes, which utilize reduced tetrapyrrole bilins such as phytochromobilin and PCB as chromophores^13^.

We then assumed that the PAS-GAF-FP can be truncated to the GAF domain only. However, simple removal of the PAS domain resulted in non-fluorescent protein that was highly unstable and formed visible aggregates over time. In order to stabilize the GAF domain, we aligned it with GAF domains of cyanobacteriochromes. The GAF domains of cyanobacteriochromes *An*PixJ^25^ and *Te*PixJ^26^ were shown to be stable and able to covalently bind PCB chromophore. We noticed that GAF domains of cynobacteriochromes lack a loop that serves as the knot lasso in BphPs. Therefore, we have removed it from our GAF domain too (Fig. 1A).

The resulted truncated protein containing residues 122–224, 247–315 of parental *Rp*BphP1 was weakly fluorescent. We then introduced monomerizing mutations to the carboxyl termini^3^,^5^ and performed random mutagenesis. We additionally mutated position 178 and again positions 200 and 201 (Fig. 1B). Substitutions in position 178 were found in blue-shifted two-domain iRFPs^8^. From the bacterial library of mutants we selected the brightest variants and then sequentially combined all appeared mutations in a single protein. This approach proved to be an effective method for increasing the stability of proteins^8^. The brightest variant was termed GAF-FP. Overall GAF-FP had 25 amino acid residue substitutions, i.e. ~15% of the whole protein, and additionally lacked 22 residues of the knot loop.

The similarity of the engineered GAF-FP with the PCB-binding GAF domains of cyanobacteriochromes suggested that GAF-FP could incorporate both BV and PCB. In nature and in engineered bacteria, PCB is produced from BV by enzymatic reduction reaction. As a result, PCB contains two double bonds less than BV, making its protein adduct spectrally blue-shifted (Supplementary Fig. 1).

### Spectral characterization of GAF-FP protein

**Figure 2:**
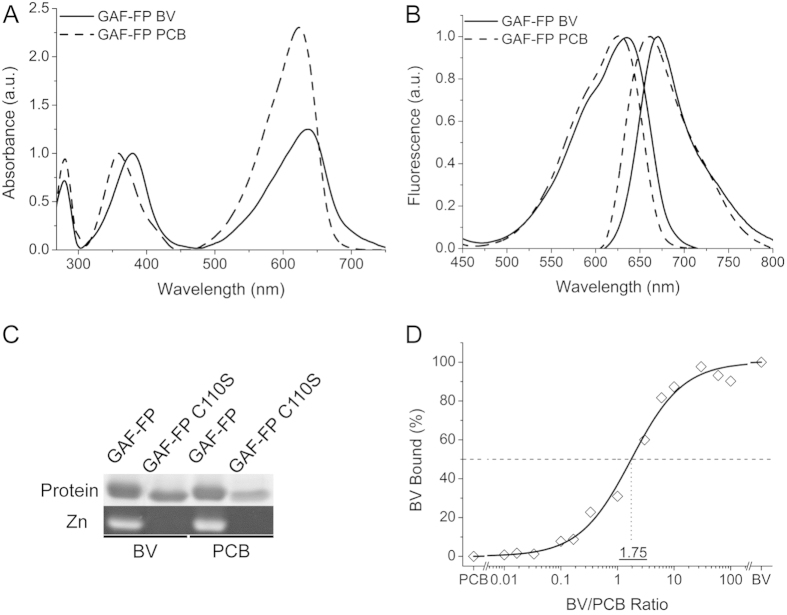
Spectral properties of GAF-FP with BV and PCB chromophores. (**A**) Absorbance spectra of GAF-FP bound with either BV or PCB chromophores normalized to absorbance at Soret band. (**B**) Normalized excitation and emission spectra of GAF-FP with either BV or PCB. (**C**) Assay of covalent BV or PCB binding to GAF-FP and GAF-FP/C110S mutant. Protein staining and zinc stating are shown. (**D**) BV versus PCB competition assay for binding to GAF-FP apoprotein.

We characterized spectral properties of GAF-FP. Overall, spectra of GAF-FP were similar to those of other BphP-based NIR FPs, indicating that GAF-FP efficiently incorporated both chromophores^3^,^4^,^5^,^6^,^7^,^8^. The GAF-FP has two absorption bands in a visible range: Soret band at 359 nm/379 nm and Q-band at 623 nm/637 nm for the PCB/BV chromophore-containing forms, respectively (Fig. 2A). Excitation spectra of GAF-FP follow the shape of Q-band absorption and have maxima at 625 nm/635 nm for the PCB/BV forms. Emission spectra have the Stokes shift of about 35 nm, resulting in fluorescence maxima at 657 nm/670 nm, respectively (Fig. 2B). The molar extinction coefficient and fluorescence quantum yield were 49,800 M^−1^cm^−1^/80,500 M^−1^cm^−1^ and 0.073/0.121 for GAF-FP with BV/PCB, respectively (Table 1).

To test if binding of chromophores to GAF-FP occurs via Cys in the GAF domain (Cys110, numbering follows that of GAF-FP) covalently, we substituted this residue with Ser and applied purified protein, expressed with one of the chromophores, alongside with original GAF-FP to SDS-PAGE. The subsequent zinc staining of the gel revealed that the covalent binding of both chromophores occurs through Cys110 (Fig. 2C). The chromophore staining of both BV and PCB was not observed in the GAF-FP/Cys110Ser mutant.

To further study binding efficiency of both chromophores, we used a competition assay in which various ratios of BV and PCB within a fixed total concentration were applied to GAF-FP apoprotein (ratios ranging from 100:1 to 1:100). The ratios of BV/PCB bound were then determined spectrophotometrically from the resulting holoprotein mixtures. As shown in Fig. 2A, the absorbance spectra of the BV- and PCB-bound forms were sufficiently distinct allowing to estimate the percentage of BV and PCB bound GAF-FP forms. We found that the apoprotein bound PCB 1.75-fold more effectively than BV (Fig. 2D).

### Biochemical characterization of GAF-FP protein

**Figure 3:**
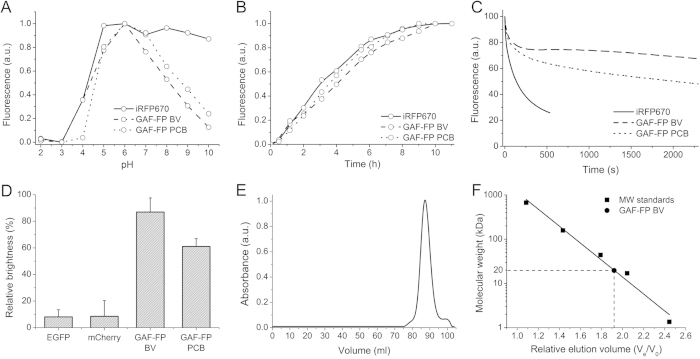
Biochemical and photochemical properties of GAF-FP. (**A**) The pH dependence of fluorescence in comparison with iRFP670 control. (**B**) Fluorescence acquisition kinetics of GAF-FP bound with either BV or PCB chromophores and of iRFP670 control at 37 °C. Time “0” corresponds to the beginning of the 1 h long pulse-chase induction of the protein expression. (**C**) Photobleaching kinetics for GAF-FP with either BV or PCB, and for iRFP670 control. The curves were normalized to absorbance spectra and extinction coefficients of FPs, spectrum of the lamp, and transmission of an excitation filter. (**D**) Efficiency of fluorescence acquisition by GAF-FP in anaerobic conditions. The ratio of a fluorescence signal of bacterial cells, expressing either GFP-like proteins (EGFP or mCherry) or GAF-FP with either BV or PCB, after 24 h in anaerobic condition to a fluorescence signal developed after subsequent 24 h maturation of these FPs in presence of oxygen. Error bars, s.d. (n = 3). (**E**) Size-exclusion chromatography of GAF-FP bound with BV. (**F**) Size exclusion chromatography calibration plot. GAF-FP peak corresponds to V_e_/V_o_ value of 1.9. V_e_, elution volume; V_0_, void volume of the column.

We next characterized biochemical properties of GAF-FP. GAF-FP had two apparent p*K*a values (Fig. 3A), of 4.0/4.6 in acid region and of 7.8/8.3 in alkaline for the BV/PCB forms, respectively. The pH changes did not cause a shape change of the excitation and emission spectra while titrating GAF-FP in the direction of either alkaline or acidic pH values from the maximum molecular brightness at pH 6.0 (Supplementary Fig. 2). At physiological pH of 7.2 GAF-FP exhibited 70% of its maximal fluorescence brightness.

Then we compared folding and chromophore incorporation kinetics of GAF-FP with two-domain NIR FP having similar spectral properties, iRFP670^8^. For this we monitored fluorescence in bacteria upon pulse-chase induction of the protein expression. GAF-FP with any of chromophores had a half-time of fluorescence acquisition of 3.5–4.0 h, which is similar to that of iRFP670 (Fig. 3B). The study of GAF-FP photostability revealed its significant resistance to photobleaching in comparison to that of iRFP670 (Fig. 3C). GAF-FP had the complex photobleaching kinetics with two components, the first photobleached within ~200 s, reaching of 80% of initial fluorescence level, while the second remained fluorescent throughout long light exposures (>2,000 s).

To study whether GAF-FP is capable to acquire fluorescence in anaerobic conditions we compared fluorescence in bacteria upon pulse-chase induction of protein expression in anaerobic condition to level of fluorescence in the presence of oxygen. In comparison with common GFP-like proteins, GAF-FP reached up to 90%/60% of maximum level of fluorescence for BV/PCB bound forms, respectively, while EGFP and mCherry fluorescence in anaerobic conditions did not exceed 10% of that in aerobic ones (Fig. 3D).

Oligomeric state of GAF-FP studied by size exclusion chromatography showed that it behaves as a monomer (Fig. 3E). The observed molecular mass of GAF-FP with a polyhistidine tag was ~20 kDa, making it 2-fold and 1.4-fold smaller than BphP-based NIR FPs and common GFP-like FPs, respectively (Fig. 3D).

**Figure 4:**
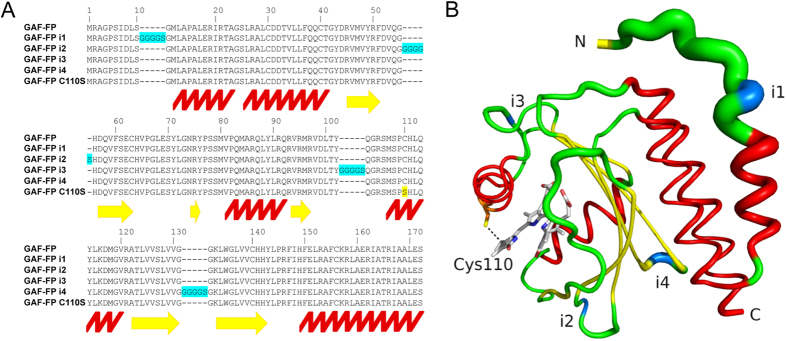
Design of insertion variants of GAF-FP. (**A**) Alignment of the amino acid sequences of GAF-FP with GAF-FP i1, GAF-FP i2, GAF-FP i3, GAF-FP i4 and GAF-FP Cys110Ser templates. Insertions in GAF-FP are highlighted in blue, and Cys110Ser mutation is highlighted in yellow. Positions of α-helices are marked with the red zig-zag line, and positions of β-sheets are marked with the yellow arrows. Amino acid numbering follows that of GAF-FP without inserts. (**B**) Structural organization of GAF-FP based on the structure of GAF domain of parental *Rp*BphP1^23^. The thickness of the line indicates the magnitude of the structural β-factor. The α-helices colored in red, β-sheets are colored in yellow, and positions of the inserts are colored in blue.

To characterize stability of GAF-FP structure, we randomly introduced a small peptide insertion of five amino acids in the protein sequence. From the library of the insertion-containing mutants, we selected the GAF-FP variants that retained high fluorescence. The four brightest variants were assigned with indexes according to the position of the insert in the amino acid sequence of GAF-FP (Fig. 4A). No significant variations in spectral and biochemical properties were found in the GAF-FP insertion variants (Supplementary Table 1). We mapped positions of the inserts on the structure of the GAF domain of *Rp*BphP1 (Fig. 4B). All inserts were located in regions without secondary structure and with relatively high flexibility, suggesting that GAF-FP predominantly retains structure of the wild-type GAF domain and exhibits rather high stability.

### Expression of GAF-FP in mammalian cells

**Figure 5:**
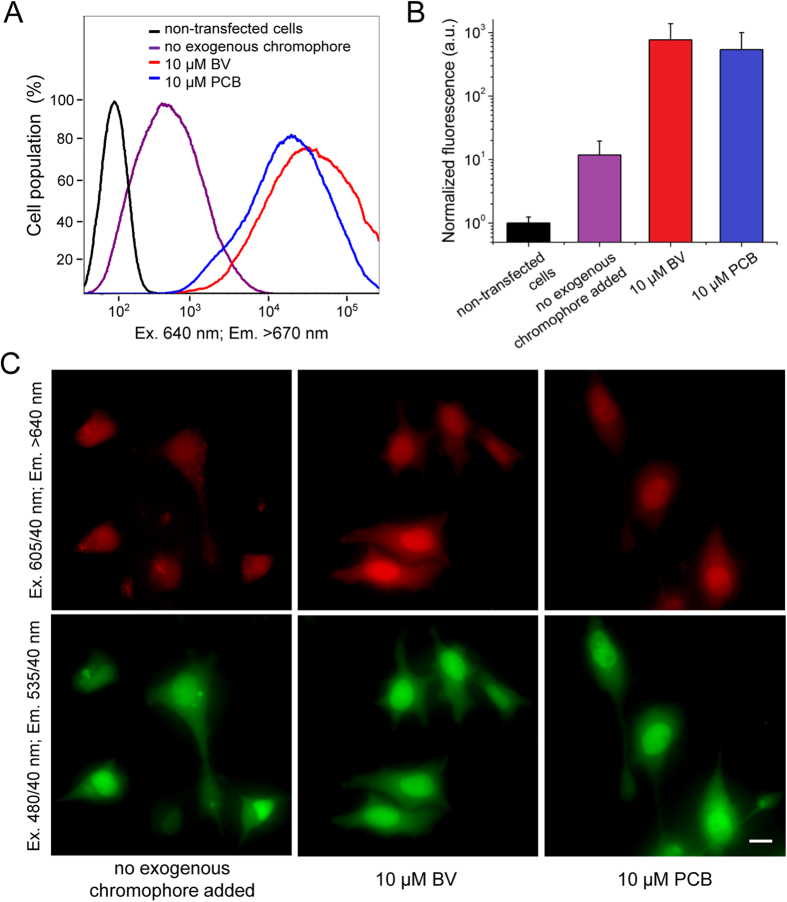
Expression of GAF-FP in mammalian cells. (**A**) Flow cytometry histograms of live HeLa cells transiently transfected with the GAF-FP—sfGFP construct with and without addition of 10 μM BV or PCB. Only sfGFP positive cells were analyzed. The NIR fluorescence was excited by the 640 nm laser and detected using the 670 nm LP emission filter. (**B**) NIR fluorescence signal of live HeLa cells detected by flow cytometry and normalized to absorbance of the respective protein at the excitation wavelength and to overlap of the fluorescence spectrum of the protein with the transmission of the emission filter. Error bars, s.d. (n = 3). (**C**) NIR and green fluorescence images of live HeLa cells transiently transfected with the GAF-FP—sfGFP construct with and without addition of 10 μM BV or PCB. Acquisition time in the NIR channel for the left image was 6.5-fold larger than for the middle and left images. Scale bar, 20 μm.

To characterize GAF-FP in live mammalian cells, we used fluorescence-activated flow cytometry and epifluorescence microscopy. To facilitate detection of transfected cells we fused GAF-FP with superfolder GFP (sfGFP)^27^ using a flexible linker of 20 amino acids. The transiently transfected live HeLa cells were analyzed 48 h after the transfection with and without 10 μM BV or PCB (Fig. 5A). The GAF-FP expressing HeLa cells, without addition of the exogenous chromophore, were 12-fold brighter than the non-transfected cells. The cell treatment with BV and PCB for 24 h before the analysis increased their fluorescence brightness relative to the untreated GAF-FP expressing cells 65- and 45-fold, respectively (Fig. 5B). Epifluorescence microscopy of the transiently transfected live HeLa cells showed evenly distributed over the whole cells NIR fluorescence signal without any aggregate formation (Fig. 5C).

### Near-infrared luciferase based on GAF-FP fusion

To explore the monomeric state, small size and ability of GAF-FP to absorb light in Soret band we decided to develop a bioluminescent NIR probe. For this, we designed several fusion constructs consisting of GAF-FP and enhanced luciferase mutant, called *R*Luc8, from *Renilla reniformis*. *R*Luc8 with a methoxy-coelenterazine-methoxy substrate emits light at ~400 nm, which has substantial overlap with Soret band of GAF-FP containing BV (Supplementary Fig. 3). In the chimeric construct, energy from the oxidized by *R*Luc8 substrate migrates via bioluminescence resonance energy transfer through Soret band to GAF-FP and then is emitted in GAF-FP NIR maximum at 670 nm. We tested several constructs varying an order of both proteins and a length of the linker between them (Supplementary Fig. 4). The best construct, GAF-FP—*R*Luc8 had the FP at the N-terminus and the luciferase at the C-terminus of chimeric protein, and a short linker of two amino acids.

**Figure 6:**
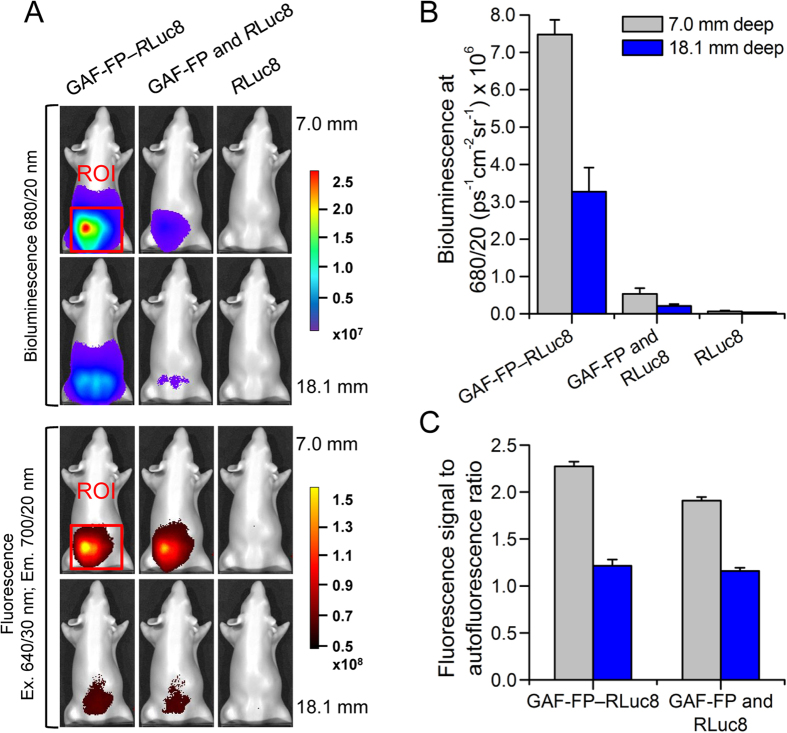
Performance of GAF-FP and GAF-FP—*R*Luc8 chimera in model deep-tissue imaging. (**A**) Bioluminescence and fluorescence signals of the chimeric protein, the GAF-FP and *R*Luc8 mixture or *R*Luc8 alone in a phantom mouse. The color bars indicate the total bioluminescence radiance (photons s^−1^ cm^−2^ steradian^−1^) or the total fluorescence radiant efficiency (photons s^−1^ cm^−2^ steradian^−1^ per μW cm^−2^). (**B**) Values of the bioluminescence signals for the images in (**A**). (**C**) Values of the fluorescence signal-to-autofluorescence background ratios for the images in (**A**). Error bars, s.d. (n = 3).

To evaluate the performance of GAF-FP—*R*Luc8 in comparison with other bioluminescent probes, we used a phantom mouse having the absorbance, light-scattering properties and autofluorescence matching those of natural mouse tissues. Equal amounts of purified GAF-FP—*R*Luc8, mixture of GAF-FP and *R*Luc8 or *R*Luc8 only were mixed with the substrate and imaged in the phantom at two depths of 7.0 mm and 18.1 mm in different spectral channels. We quantified bioluminescence intensities and ratios of the fluorescence signal to autofluorescence background. In 680/20 nm band GAF-FP—*R*Luc8 had more than 10-fold higher signal at both depths in comparison with the GAF-FP and *R*Luc8 mixture and more than 100-fold higher signal in comparison with *R*Luc8 alone (Fig. 6A,B). GAF-FP also had high ratio of fluorescence to autofluorescence (Fig. 6A,C). For GAF-FP—*R*Luc8 at 7.0 mm and 18.1 mm depth ratios were 2.3 and 1.2, respectively.

## Summary

We have developed the protein engineering approach, which has resulted in the smallest NIR FP reported so far. Introduction of the Cys residue into the GAF domain^22^ allowed us to truncate the two-domain FP to only a single chromophore-binding domain and to remove BphP conservative knot structure. GAF-FP exhibits a series of the advanced properties, such as monomeric behavior, high photostability, ability to fluoresce in anaerobic conditions, and tolerance to peptide insertions. Moreover, the ability to bind two distinct chromophores makes GAF-FP suitable for applications in various biological hosts producing different endogenous tetrapyrroles.

Further studies should include the improvement of GAF-FP brightness and stability in mammalian cells, as well as the design of spectrally distinct single-domain NIR FPs. The promising templates for the described here molecular evolution could be various BphPs, such as *Rp*BphP2, *Rp*BphP6 and *Dr*BhpP, which already served for engineering of two-domain NIR FPs.

The engineered chimera with *R*Luc8 luciferase converted GAF-FP into the NIR luciferase with multimodal (fluorescence and bioluminescence) imaging properties. Thereby, GAF-FP—*R*Luc8 chimera adds a new color to the pallet of the available cyan, yellow and orange chimeric luciferases^28^. This approach can be also applied to other NIR FPs, utilizing tetrapyrrole chromophores and thereby having the Soret absorption band. Overall, the rational design of GAF-FP described here provides the guidelines for engineering of advanced small NIR FPs and chimeric NIR luciferases.

## Methods

### Mutagenesis and screening of libraries of mutants

A PCR-amplified *BglII*-*EcoRI* fragments encoding *Rp*BphP1 PAS-GAF domains (first 315 amino acids) or only GAF domain (amino acids from 122 to 315) was cloned into the pBAD/His B vector (Life Technologies/Invitrogen). Site-specific mutagenesis was performed using a QuikChange Mutagenesis Kit (Stratagene). Random mutagenesis was performed using a GeneMorph II Random Mutagenesis Kit (Stratagene) under coditions resulting in a mutation frequency of up to 16 mutations per 1,000 base pairs. After mutagenesis, a mixture of mutated genes was electroporated into *Escherichia coli* strain LMG194 host cells (Life Technologies/Invitrogen) containing the pWA23h plasmid^8^ facilitating biliverdin synthesis via co-expression of heme oxygenase.

Typical mutant libraries consisted of about 10^6^–10^8^ independent clones. The LMG194 cells were grown overnight at 37 °C in RM minimal medium supplemented with ampicillin and kanamycin. Protein expression in the libraries was induced with 0.004% arabinose and 0.04% rhamnose. The cells were grown for 6–8 h at 37 °C, then at 30 °C for 10–12 h, and at last at 18 °C for 24 h. Before flow cytometry screening, bacterial cells were washed with phosphate-buffered saline (PBS) and diluted with PBS to an optical density of 0.03 at 600 nm. The libraries were screened using MoFlo XDP fluorescence-activated cell sorter equipped with argon, krypton and He-Ne gas lasers (Beckman Coulter). Typically about ten sizes of each library were sorted using a 633 nm laser for excitation and a 647 nm LP emission filter for positive selection.

The brightest collected far-red bacterial cells were rescued in SOC medium at 37 °C for 1 h and then grown on LB/ampicillin/kanamycin Petri dishes supplemented with 0.004% arabinose, 0.02% rhamnose, overnight at 37 °C and then incubated at 18 °C for 24 h. The dishes were analyzed with a Leica MZ16F fluorescence stereomicroscope equipped with 605/40 nm or 650/45 nm excitation and 640 nm LP or 690 nm LP emission filters (Chroma) and a CCD camera. Approximately 20–50 clones in each screen were selected, their DNA was sequenced. A mixture of several selected mutants was then used as a template for the next round of mutagenesis.

### Spectral and photochemical characterization

The GAF-FP, its mutants with polyhistidine tags on the N termini were expressed in LMG194 bacterial cells containing the pWA23h plasmid (or pPL-PCB plasmid facilitating phycocyanobilin synthesis^29^) grown in RM medium supplemented with ampicillin, kanamycin, 0.004% arabinose and 0.02% rhamnose for 6–8 h at 37 °C, then at 30 °C for 10–12 h, and at last at 18 °C for 24 h. Proteins were purified using Ni-NTA agarose (Qiagen) according to the manufacturer’s protocol with minor modification. Solutions used for purification of the protein contained additional 0.6 mM of sodium chloride and the wash buffer contained 100 mM EDTA instead of 400 mM imidazole. Fluorescence spectra were recorded using a FluoroMax-3 spectrofluorometer (Jobin Yvon). A Hitachi U-2000 spectrophotometer was used for absorbance measurements. Background light scattering was removed by subtracting a fitted λ^4^ curve from the measured spectrum.

The extinction coefficient was calculated from a comparison of absorbance values at the main peak at around 630 with the absorbance value at the smaller peak at around 380 nm, assuming the latter had an extinction coefficient of free BV of 39,900 M^−1^ cm^−1^, or an extinction coefficient of free PCB of 35,500 M^−1^cm^−1^ (ref. 30). To determine fluorescence quantum yield, we compared the fluorescence signal of a purified protein to that of an equally absorbing Nile blue dye. pH titrations were done using a series of Hydrion buffers (Micro Essential Laboratory).

To evaluate GAF-FP photostability, the LMG194 bacterial cells containing target protein in pBAD/His B plasmid (GAF-FP or iRFP670) and pWA23h or pPL-PCB plasmid were grown on LB/ampicillin/kanamycin Petri dishes supplemented with 0.004% arabinose, 0.02% rhamnose overnight at 37 °C. Bacteria were photobleached using an Olympus IX81 inverted epifluorescence microscope equipped with a 200 W Me-Ha arc lamp (Lumen220Pro, Prior), 100 × 1.4 NA oil-immersion objective lens (UPlanSApo, Olympus) and 605/40 nm (exciter) and 640 nm (LP emitter) filter set (Chroma). SlideBook v.4.1 software (Intelligent Imaging Innovations) was used to operate the Olympus IX81 inverted microscope. Raw data were normalized to absorbance spectra and extinction coefficients of the proteins, the spectrum of 200 W Me-Ha arc lamp and the transmission of 605/40 nm photobleaching filter.

### Biochemical characterization

To study protein folding and maturation in cells, we grew the LMG194 bacterial cells containing target protein in pBAD/His B plasmid (GAF-FP or iRFP670) and pWA23h or pPL-PCB plasmid overnight at 37 °C in RM medium. The next morning, 0.2% rhamnose was added for 2 h, subsequently 0.002% arabinose was added, and cells were cultured for 1 h. Then arabinose was washed out, and cells were cultured in RM medium with 0.2% rhamnose at 37 °C. Fluorescence intensities of the equal aliquots of the cell suspension were measured at intervals after dilution to the same optical density of 0.2, and the obtained values were multiplied by the dilution factor. For EGFP expression, the procedure was the same except that the LMG194 strain did not contain plasmids for bilin synthesis.

Bilin binding was assayed by zinc-induced fluorescence of the chromoprotein following SDS-PAGE. To assess chromophore preference, the apoprotein was expressed as above without simultaneous chromophore synthesis and purified as described above. Then we used the procedure described previously^31^. The apoprotein was assembled with the chromophore *in vitro*, using a >10-fold molar excess of either BV, PCB or various ratios of BV and PCB. Absorption spectra were obtained. To quantitate binding efficiency of BV relative to PCB, the absorbance maxima of BV- and PCB-bound proteins were identified in the Q-band for the spectrum generated by each BV/PCB mixture. By incorporating these absorbance values in the ratio Q_BV_/Q_PCB_, the relative contributions of BV-bound and PCB-bound holoproteins in the mixtures were calculated. These ratios were normalized by subtracting the value calculated for the 100% PCB sample (100% PCB-bound sample now represented 0% BV incorporation) from all other values and scaling all of the adjusted values so that the values from samples generated solely with BV equaled 100%.

To perform size exclusion liquid chromatography a 2 ml volume of purified GAF-FP was applied on the HiLoad 16/600 Superdex 200 column (GE Healthcare) equilibrated with 10 mM Hepes buffer pH 7.4 containing 50 μM EDTA, 10% glycerol, 150 mM NaCl, 1 mM DTT, 0.2 mM PMSF, 0.01% EP-40 and 0.2 mM benzodiazepin. A 1 ml/min flow rate was used. The column was calibrated using the gel filtration standards from Bio-Rad.

For the anaerobic protein maturation assay the LMG194 bacterial cells containing pBAD/HisB-GAF-FP and either pWA23h or pPL-PCB plasmids were grown on LB/ampicillin/kanamycin Petri dishes supplemented with 0.02% rhamnose overnight at 37 °C. Before dishes were placed in the chamber of anaerobic gas generating system (Mitsubishi gas chemical) for 24 h at 37 °C, bacterial streaks were sprayed with 200 μl of 20% arabinose solution. The dishes were analyzed with the Leica MZ16F fluorescence stereomicroscope equipped with 480/40 nm, 570/30 nm and 605/40 nm excitation, and 530/40 nm, 615/40 nm, 640 nm LP or 690 nm LP emission filters (Chroma) and a CCD camera. Immediately after analysis Petri dishes were transferred to 4 °C for additional 48 h incubation in the presence of oxygen. During this time only already synthetized FP will be able to bind chromophore, while the synthesis of new protein will be significantly slowed-down. The dishes were analyzed again, level of fluorescence after incubation at 4 °C was considered to be 100%. According to that estimation we found the value of relative protein maturation of GAF-FP in anaerobic conditions. For EGFP and mCherry expression, the procedure was the same except that the LMG194 strain did not contain plasmids for bilin synthesis. All quantitative measurements of fluorescence signal for bacteria were performed using the ImageJ software^32^.

### Expression in mammalian cells

A PCR-amlified *Age*I*-Kpn*I fragment encoding GAF-FP and *Kpn*I*-Not*I fragment encoding sfGFP^26^ were swapped with a gene encoding EGFP in a pEGFP-N1 vector (Clontech), resulting in pGAF-FP-sfGFP-N1 plasmid. HeLa cells were grown in DMEM medium supplemented with 10% FBS, 0.5% penicillin-streptomycin and 2 mM glutamine (Life Technologies/Invitrogen). For epifluorescence microscopy, cells were cultured in 35 mm glass-bottom Petri dishes with no. 1 coverglass (MatTek). Plasmid transfections were performed using an Effectene reagent (Qiagen). If necessary, tetrapyrroles were added to cell culture for 24 h before imaging.

### Flow cytometry

Flow cytometry analysis was performed using a BD LSRII flow cytometer. To detect live HeLa cells transfected with the pGAF-FP-sfGFP-N1 plasmid, a 488 nm Ar gas laser line, a 640 nm solid-state laser, and the 530/40 nm and 670 nm long pass emission filters were used. To quantify the NIR fluorescence brightness of the transiently transfected cells, the mean NIR fluorescence signal was normalized to the absorbance spectra and the extinction coefficients of GAF-FP and to the transmission of the 670 nm long pass emission filter. The histograms of cell populations and mean NIR fluorescence intensities of the analyzed cells were obtained using the FCS express v.3 software (DeNovo Software).

### Fluorescence microscopy

Epifluorescence microscopy of live HeLa cells was performed 24 h after the transfection. HeLa cells were imaged using an Olympus IX81 inverted epifluorescence microscope equipped with a 200 W Me-Ha arc lamp (Lumen220 Pro; Prior), a 40× 0.65–1.35 NA oil objective lens (UPlanSApo; Olympus), and two filter sets (480/40 nm excitation and 535/40 nm emission; 605/40 nm excitation and 640 nm long pass emission) (Chroma). SlideBook v. 4.1 software (Intelligent Imaging Innovations) was used to operate the microscope.

### Construction and characterization of luciferase proteins

A PCR-amlified *Bgl*II*-Kpn*I fragment encoding GAF-FP and *Kpn*I*-EcoR*I fragment encoding *Renilla* luciferase 8 (*R*Luc8)^33^ (Addgene plasmid #51970) were cloned into the pBAD/His B vector (Life Technologies/Invitrogen). For cloning of fragments in reverse order a PCR-amplified *Bgl*II*-Kpn*I fragment encoding *R*Luc8 and *KpnI*-*EcoR*I fragment encoding GAF-FP were used. Primers with a *KpnI* restriction site at the end encoded the linker between proteins. The linker consisted of two amino acids (-GT-), derived from *Kpn*I restriction site, 7 amino acids (-GGGGSGT-) or 14 amino acids (-GGGGSGTGGGGSGG-). The amino acid composition of the linker was chosen so that it has a high hydrophilicity and flexibility. A PCR-amplified *Bgl*II*-EcoR*I fragment encoding *R*Luc8 was also cloned in pBAD/His B vector.

To compare the level of NIR signal between chimeric fusion constructs, we grew LMG194 bacterial cells overnight at 37 °C in RM minimal medium supplemented with ampicillin, kanamycin, 0.004% arabinose and 0.04% rhamnose. Further the suspensions were diluted to the same optical density of 0.2 at 600 nm. Luminescent intensities in 680/20-nm channel of equal aliquots of the cell suspensions were measured immediately after mixing with methoxy-coelenterazine-methoxy (Me-O-CTZ-O-Me; NanoLight Technologies) to achieve a final concentration of 50 μM according to the manufacturer’s recommendations. Images were taken in the luminescence mode of the IVIS Spectrum (PerkinElmer). All quantitative measurements of luminescence signal were performed using the Living Image v.4.3.1 software (PerkinElmer).

### Protein imaging in phantom mouse

Recombinant *R*Luc8 was expressed in LMG194 bacterial cells and then purified using Ni-NTA agarose (Qiagen). The purified chimeric protein, GAF-FP and *R*Luc8 were diluted to equal concentrations of 8 μM, calculated from the extinction coefficients at the chromophore absorbance maxima or at 280 nm. A 5 μl volume of each protein or their mixture was mixed with aliquot of methoxy-coelenterazine-methoxy substrate (final concentration of 50 μM). Immediately thereafter, the samples were placed inside into one of two available bores in an XFM-2 phantom mouse (PerkinElmer/Caliper) and images were taken. The bores were located at depth of 7.0 mm and 18.1 mm from the imaging surface. Images were taken in the luminescence or epifluorescence mode of the IVIS Spectrum (PerkinElmer). For the best quality images we tested different combinations of the available excitation and emission channels in the range from 500 to 750 nm. To register bioluminescence we used 680/20 nm filter. To register fluorescence we used 640/20 nm excitation and 700/20 nm emission filters; the phantom mouse without protein sample inside was used as a background reference. All quantitative measurements of luminescence or fluorescence signal as well as mathematical operations with images were performed using the Living Image v.4.3.1 software (PerkinElmer).

## Additional Information

**How to cite this article**: Rumyantsev, K. A. *et al.* Minimal domain of bacterial phytochrome required for chromophore binding and fluorescence. *Sci. Rep.* **5**, 18348; doi: 10.1038/srep18348 (2015).

## Supplementary Material

Supplementary Information

## Figures and Tables

**Table 1:** Characteristics of GAF-FP protein with BV and PCB chromophores measured at pH 7.2 and room temperature.

Fluorescent protein	Excitation maximum (nm)	Emission maximum (nm)	Extinction coefficient (M^−1^ cm^−1^)	Fluorescence quantum yield (%)	Molecular brightness (%)	Maturation t_50%_ at 37 ^o^C (h)	Photostability t_80%_ (s)	p*K*a_1_	p*K*a_2_
GAF-FP BV	635	670	49,800	7.3	100	4.0	97	4.0	7.8
GAF-FP PCB	625	657	80,500	12.1	268	3.5	64	4.6	8.3

## References

[b1] Shcherbakova D. M., Baloban M. & Verkhusha V. V. Near-infrared fluorescent proteins engineered from bacterial phytochromes. Current Opinion in Chemical Biology 27, 52–63 (2015). 26115447 10.1016/j.cbpa.2015.06.005 PMC4553112

[b2] Piatkevich K. D., Subach F. V. & Verkhusha V. V. Engineering of bacterial phytochromes for near-infrared imaging, sensing, and light-control in mammals. Chemical Society reviews 42, 3441–3452 (2013). 23361376 10.1039/c3cs35458j PMC3618476

[b3] Shu X. *et al.* Mammalian expression of infrared fluorescent proteins engineered from a bacterial phytochrome. Science 324, 804–807 (2009). 19423828 10.1126/science.1168683 PMC2763207

[b4] Yu D. *et al.* An improved monomeric infrared fluorescent protein for neuronal and tumour brain imaging. Nature communications 5, 3626 (2014). 10.1038/ncomms4626 PMC4077998 24832154

[b5] Auldridge M. E., Satyshur K. A., Anstrom D. M. & Forest K. T. Structure-guided engineering enhances a phytochrome-based infrared fluorescent protein. The Journal of biological chemistry 287, 7000–7009 (2012). 22210774 10.1074/jbc.M111.295121 PMC3293566

[b6] Bhattacharya S., Auldridge M. E., Lehtivuori H., Ihalainen J. A. & Forest K. T. Origins of fluorescence in evolved bacteriophytochromes. The Journal of biological chemistry 289, 32144–32152 (2014). 25253687 10.1074/jbc.M114.589739 PMC4231690

[b7] Filonov G. S. *et al.* Bright and stable near-infrared fluorescent protein for *in vivo* imaging. Nature biotechnology 29, 757–761 (2011). 10.1038/nbt.1918 PMC3152693 21765402

[b8] Shcherbakova D. M. & Verkhusha V. V. Near-infrared fluorescent proteins for multicolor *in vivo* imaging. Nature methods 10, 751–754 (2013). 23770755 10.1038/nmeth.2521 PMC3737237

[b9] Piatkevich K. D., Subach F. V. & Verkhusha V. V. Far-red light photoactivatable near-infrared fluorescent proteins engineered from a bacterial phytochrome. Nature communications 4, 2153 (2013). 10.1038/ncomms3153 PMC3749836 23842578

[b10] Filonov G. S. & Verkhusha V. V. A near-infrared BiFC reporter for *in vivo* imaging of protein-protein interactions. Chemistry & biology 20, 1078–1086 (2013). 23891149 10.1016/j.chembiol.2013.06.009 PMC3757571

[b11] Chen M. *et al.* Novel near-infrared BiFC systems from a bacterial phytochrome for imaging protein interactions and drug evaluation under physiological conditions. Biomaterials 48, 97–107 (2015). 25701035 10.1016/j.biomaterials.2015.01.038

[b12] Bhoo S. H., Davis S. J., Walker J., Karniol B. & Vierstra R. D. Bacteriophytochromes are photochromic histidine kinases using a biliverdin chromophore. Nature 414, 776–779 (2001). 11742406 10.1038/414776a

[b13] Rockwell N. C. & Lagarias J. C. A brief history of phytochromes. ChemPhysChem 11, 1172–1180 (2010). 20155775 10.1002/cphc.200900894 PMC2880163

[b14] Wagner J. R., Brunzelle J. S., Forest K. T. & Vierstra R. D. A light-sensing knot revealed by the structure of the chromophore-binding domain of phytochrome. Nature 438, 325–331 (2005). 16292304 10.1038/nature04118

[b15] Stepanenko O. V. *et al.* A knot in the protein structure - probing the near-infrared fluorescent protein iRFP designed from a bacterial phytochrome. The FEBS journal 281, 2284–2298 (2014). 24628916 10.1111/febs.12781 PMC4009348

[b16] Mishin A. S., Belousov V. V., Solntsev K. M. & Lukyanov K. A. Novel uses of fluorescent proteins. Current Opinion in Chemical Biology 27, 1–9 (2015). 26022943 10.1016/j.cbpa.2015.05.002

[b17] Lamparter T. Evolution of cyanobacterial and plant phytochromes. FEBS letters 573, 1–5 (2004). 15327965 10.1016/j.febslet.2004.07.050

[b18] Fischer A. J. *et al.* Multiple roles of a conserved GAF domain tyrosine residue in cyanobacterial and plant phytochromes. Biochemistry 44, 15203–15215 (2005). 16285723 10.1021/bi051633z PMC1343512

[b19] Zhang J. *et al.* Fused-gene approach to photoswitchable and fluorescent biliproteins. Angewandte Chemie 49, 5456–5458 (2010). 20583017 10.1002/anie.201001094

[b20] Rockwell N. C., Martin S. S. & Lagarias J. C. Red/green cyanobacteriochromes: sensors of color and power. Biochemistry 51, 9667–9677 (2012). 23151047 10.1021/bi3013565

[b21] Narikawa R. *et al.* A biliverdin-binding cyanobacteriochrome from the chlorophyll d-bearing cyanobacterium Acaryochloris marina. Sci Rep 5, 7950 (2015). 25609645 10.1038/srep07950 PMC4302295

[b22] Bellini D. & Papiz M. Z. Structure of a bacteriophytochrome and light-stimulated protomer swapping with a gene repressor. Structure 20, 1436–1446 (2012). 22795083 10.1016/j.str.2012.06.002

[b23] Shcherbakova D. M. *et al.* Molecular basis of spectral diversity in near-infrared phytochrome-based fluorescent proteins. Chemistry & biology 22, 1540–1551 (2015). 26590639 10.1016/j.chembiol.2015.10.007 PMC4667795

[b24] Wagner J. R. *et al.* Mutational analysis of Deinococcus radiodurans bacteriophytochrome reveals key amino acids necessary for the photochromicity and proton exchange cycle of phytochromes. The Journal of biological chemistry 283, 12212–12226 (2008). 18192276 10.1074/jbc.M709355200 PMC2431007

[b25] Narikawa R., Fukushima Y., Ishizuka T., Itoh S. & Ikeuchi M. A novel photoactive GAF domain of cyanobacteriochrome AnPixJ that shows reversible green/red photoconversion. Journal of molecular biology 380, 844–855 (2008). 18571200 10.1016/j.jmb.2008.05.035

[b26] Ishizuka T., Narikawa R., Kohchi T., Katayama M. & Ikeuchi M. Cyanobacteriochrome TePixJ of Thermosynechococcus elongatus harbors phycoviolobilin as a chromophore. Plant & cell physiology 48, 1385–1390 (2007). 17715149 10.1093/pcp/pcm106

[b27] Pedelacq J. D., Cabantous S., Tran T., Terwilliger T. C. & Waldo G. S. Engineering and characterization of a superfolder green fluorescent protein. Nature biotechnology 24, 79–88 (2006). 10.1038/nbt1172 16369541

[b28] Takai A. *et al.* Expanded palette of Nano-lanterns for real-time multicolor luminescence imaging. Proc Natl Acad Sci USA 112, 4352–4356 (2015). 25831507 10.1073/pnas.1418468112 PMC4394297

[b29] Gambetta G. A. & Lagarias J. C. Genetic engineering of phytochrome biosynthesis in bacteria. Proc Natl Acad Sci USA 98, 10566–10571 (2001). 11553807 10.1073/pnas.191375198 PMC58506

[b30] Oheocha C. Spectral properties of the phycobilins. I. Phycocyanobilin. Biochemistry 2, 375–382 (1963). 13939643 10.1021/bi00902a034

[b31] Wagner J. R., Zhang J., Brunzelle J. S., Vierstra R. D. & Forest K. T. High resolution structure of Deinococcus bacteriophytochrome yields new insights into phytochrome architecture and evolution. The Journal of biological chemistry 282, 12298–12309 (2007). 17322301 10.1074/jbc.M611824200

[b32] Schneider C. A., Rasband W. S. & Eliceiri K. W. NIH Image to ImageJ: 25 years of image analysis. Nature methods 9, 671–675 (2012). 22930834 10.1038/nmeth.2089 PMC5554542

[b33] Saito K. *et al.* Luminescent proteins for high-speed single-cell and whole-body imaging. Nature communications 3, 1262 (2012). 10.1038/ncomms2248 PMC3535334 23232392

