# Supplementary material for: Minimal domain of bacterial phytochrome required for chromophore binding and fluorescence
Source: Sci Rep. 2015 Dec 18;5:18348. doi: 10.1038/srep18348 (PMC4683375; doi:10.1038/srep18348)
Supplement: Supplementary Information [file srep18348-s1.pdf]

Minimal domain of bacterial phytochrome required for chromophore binding and fluorescence

Konstantin A. Rumyantsev, Daria M. Shcherbakova, Natalia I. Zakharova, Alexander V. Emelyanov, Konstantin K. Turoverov and Vladislav V. Verkhusha

Supplementary Materials

Supplementary Figures

Supplementary Figure 1. The structural formula of GAF-FP chromophores.

Supplementary Figure 2. The pH dependence of the normalized fluorescence excitation and emission spectra of GAF-FP bound to BV.

Supplementary Figure 3. Bioluminescence resonance energy transfer between *R*Luc8 and GAF-FP.

Supplementary Figure 4. Screening for optimal chimeric fusion construct between *R*Luc8 and GAF-FP.

Supplementary Tables

Supplementary Table 1. Spectral and biochemical properties of the GAF-FP insertion variants.

Biliverdin IXα

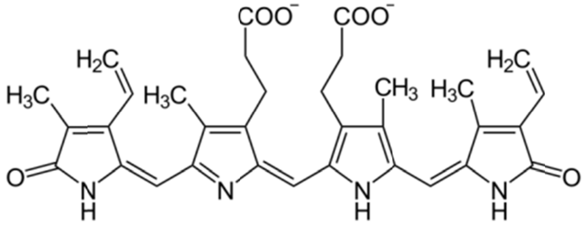

Phycocyanobilin

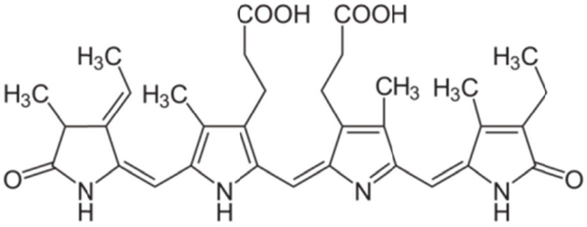

Supplementary Figure 1. The structural formula of GAF-FP chromophores. Biliverdin IXα (BV) and phycocyanobilin (PCB) are linear tetrapyrroles enzymatically derived from a heme.

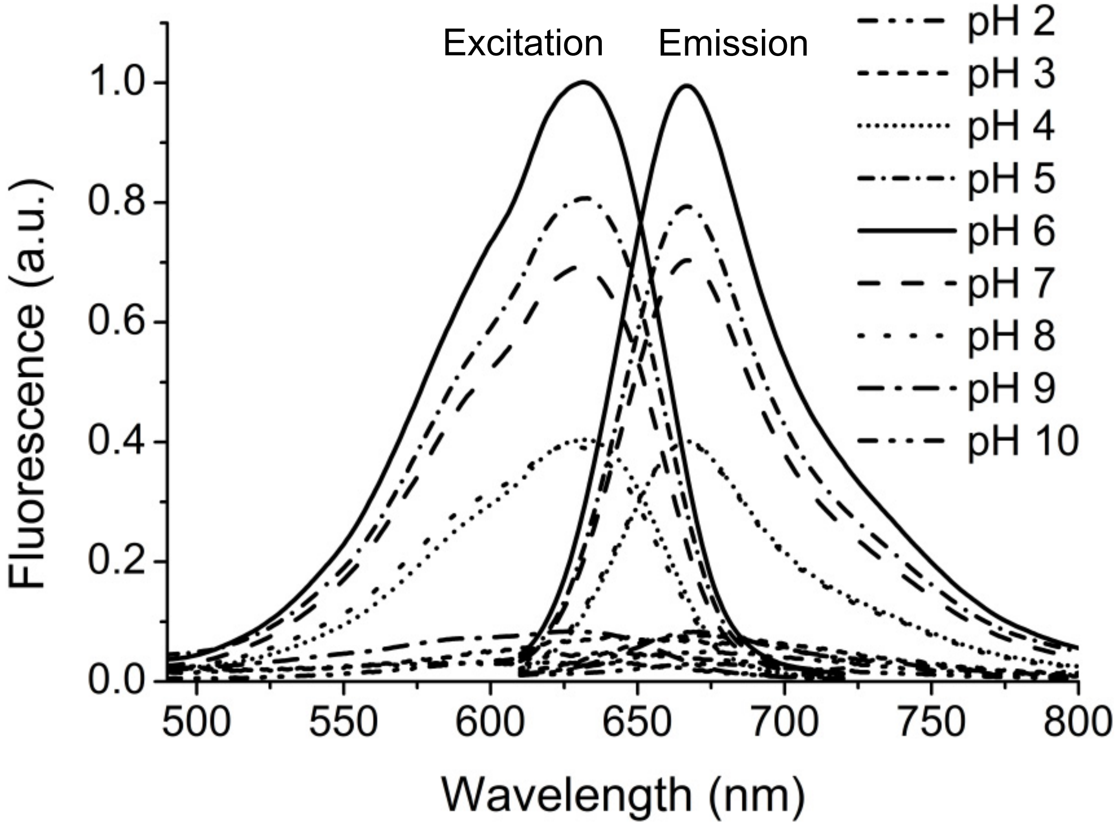

Supplementary Figure 2. The pH dependence of the normalized fluorescence excitation and emission spectra of GAF-FP bound to BV.

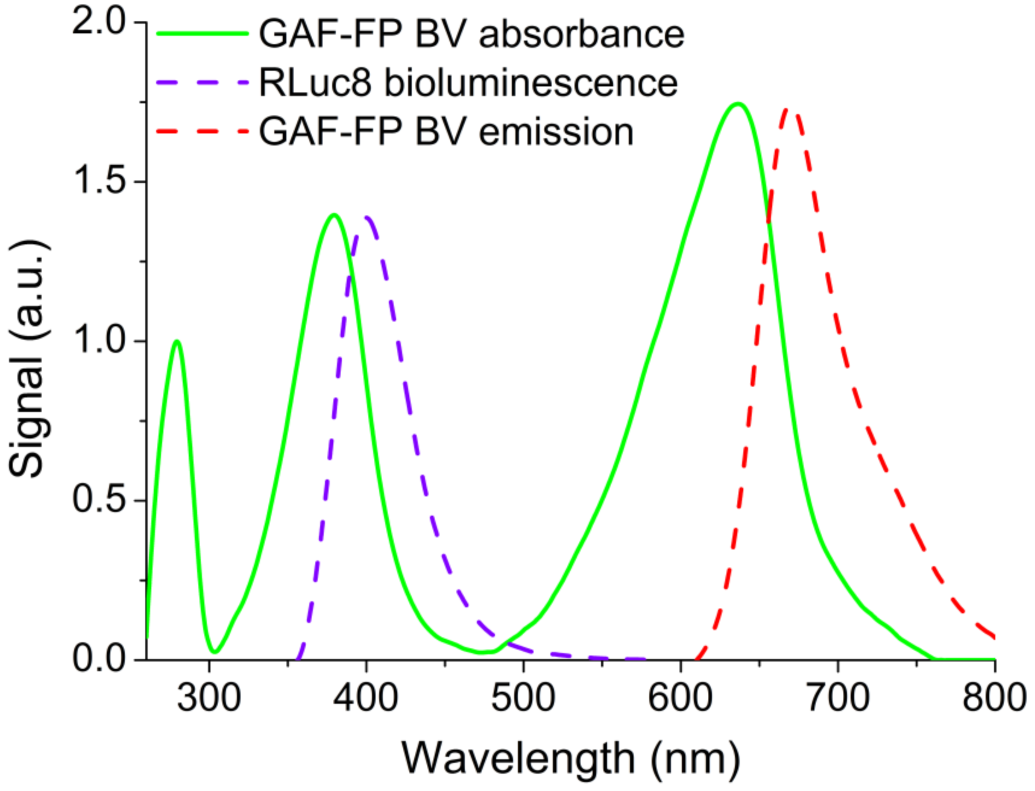

Supplementary Figure 3. Bioluminescence resonance energy transfer between *R*Luc8 and GAF-FP. Energy of the substrate oxidized by *R*Luc8 migrates via BRET to Soret band of GAF-FP. As a result, GAF-FP emits photons in its normal emission spectrum, leading to the appearance of NIR bioluminescence.

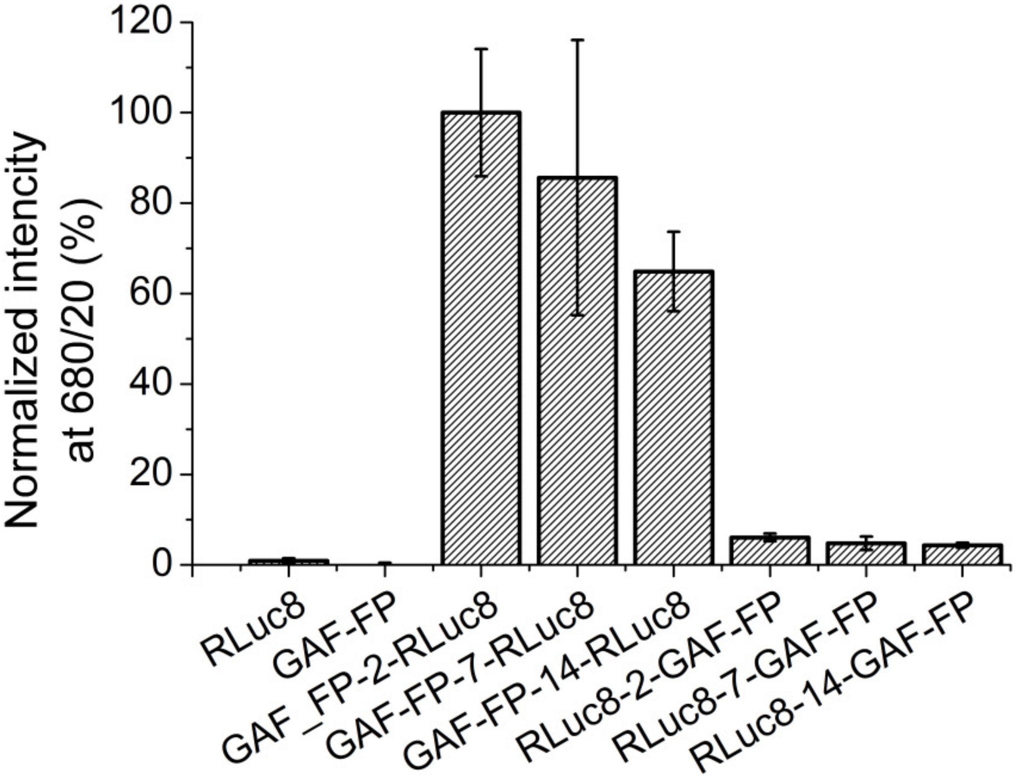

Supplementary Figure 4. Screening for optimal chimeric fusion construct between *R*Luc8 and GAF-FP. All the proteins were compared for near-infrared signal intensity when expressed in bacteria. The sample with the highest signal was taken as 100%, while the lowest as 0%. Error bars, s.d. (n = 3).

Supplementary Table 1. Spectral and biochemical properties of the GAF-FP insertion variants.

Insertion variant	Excitation maximum (nm)	Emission maximum (nm)	Extinction coefficient (M^{-1} cm^{-1})	Quantum yield (%)	Molecular brightness relative to GAF-FP BV (%)	Maturation $t_{50\%}$ at 37°C (h)	p*K*a_1	p*K*a_2	Brightness in *E. coli* at 37°C relative to GAF-FP BV (%)
GAF-FP i1 BV	635	670	45,650	5.7	72	4.1	4.8	8.1	58
GAF-FP i2 BV	635	670	52,300	6.5	94	3.7	4.7	8.1	111
GAF-FP i3 BV	635	670	49.600	6.2	85	5.4	4.7	8.0	56
GAF-FP i4 BV	635	670	47.400	5.5	72	5.1	4.6	7.8	60
GAF-FP i1 PCB	625	657	66,500	12.4	227	3.0	4.4	9.1	111
GAF-FP i2 PCB	625	657	83,000	12.7	290	3.3	4.6	8.6	182
GAF-FP i3 PCB	625	657	81,500	12.8	287	4.4	4.7	8.2	55
GAF-FP i4 PCB	625	657	72,500	11.4	227	3.7	4.8	8.4	158
